# The health outcomes and physical activity in preschoolers (HOPP) study: rationale and design

**DOI:** 10.1186/1471-2458-12-284

**Published:** 2012-04-17

**Authors:** Brian W Timmons, Nicole A Proudfoot, Maureen J MacDonald, Steven R Bray, John Cairney

**Affiliations:** 1 Child Health & Exercise Medicine Program, Department of Pediatrics, McMaster University, 1280 Main Street West, Hamilton, ON, L8S 4K1, Canada; 2 Department of Kinesiology, McMaster University, 1280 Main Street West, Hamilton, ON, L8S 4K1, Canada; 3 Departments of Family Medicine & Psychiatry and Behavioural Neurosciences, McMaster University, 1280 Main Street West, Hamilton, ON, L8S 4K1, Canada

**Keywords:** Early years, health-related fitness, body composition, cardiovascular, motor skills

## Abstract

**Background:**

The early years are the period of growth for which we know the least about the impact of physical activity. In contrast, we know that more than 90 % of school-aged Canadian children, for example, are not meeting physical activity recommendations. Such an activity crisis is a major contributor to recent trends in childhood obesity, to which preschoolers are not immune. The World Health Organization estimated that more than 42 million children under the age of 5 years were overweight world-wide in 2010. If an activity crisis exists during the preschool years, we should also be concerned about its broader impact on health. Unfortunately, the relationship between physical activity and health during the early years is poorly understood. The goal of the Health Outcomes and Physical activity in Preschoolers (HOPP) study is to describe how the prevalence and patterns of physical activity in preschoolers are associated with indices of health.

**Methods:**

The HOPP study is a prospective cohort study. We aim to recruit 400 3- to 5-year-old children (equal number of boys and girls) and test them once per year for 3 years. Each annual assessment involves 2 laboratory visits and 7 consecutive days of physical activity monitoring with protocols developed in our pilot work. At visit 1, we assess body composition, aerobic fitness, short-term muscle power, motor skills, and have the parents complete a series of questionnaires related to their child’s physical activity, health-related quality of life and general behaviour. Over 7 consecutive days each child wears an accelerometer on his/her waist to objectively monitor physical activity. The accelerometer is programmed to record movement every 3 s, which is needed to accurately capture the intensity of physical activity. At visit 2, we assess vascular structure and function using ultrasound. To assess the associations between physical activity and health outcomes, our primary analysis will involve mixed-effects models for longitudinal analyses.

**Discussion:**

The HOPP study addresses a significant gap in health research and our findings will hold the potential to shape public health policy for active living during the early years.

## Background

Even though early childhood is a critical period for the development of active living behaviours, it is the period of growth for which we know the least about the health impact of physical activity [1]. In contrast, the health benefits of physical activity for school-aged children are well established [2,3]. It may be that society has traditionally thought of the early years as a time when very young children are ‘active enough’, so important questions regarding physical activity and health in this age group have gone unanswered.

In Canada and around the world, preschoolers are not immune to trends in obesity. Canadian studies estimate the prevalence of obesity among 2- to 5-year-old children to be ~8% [4,5] and ~11% [6], depending on geographical location, and the World Health Organization estimated that in 2010 more than 42 million children under the age of 5 years were overweight worldwide [7]. One consequence of obesity during the early years may be an acceleration of what has previously been considered adult-onset disease. We know, for example, that signs of atherosclerosis are present in early childhood [8], and reduced vascular function has been identified as part of the atherosclerotic process. There is also evidence that risk of cardiovascular disease later in life can be attributed to health-related fitness levels during childhood [9]. Health-related fitness denotes characteristics that are related to the prevention of disease or the promotion of health [10], such as aerobic fitness. Very little is known about health-related fitness during the early years. Thus, an inactive lifestyle, including low fitness, combined with excess adiposity during the preschool years may accelerate the development of cardiovascular disease.

The need to understand the health impact of physical activity has never been greater. Studies that assess physical activity among preschoolers using objective measures (i.e., accelerometers) are usually cross-sectional in nature and typically report very low levels of physical activity and very high levels of sedentary time [1,11-13]. If we accept that contemporary preschoolers have low levels of physical activity and that physical activity is needed for optimal health, then it follows that inactive preschoolers are at risk for health ‘deficits’ during the early years. However, in a previous review, we found very little evidence linking physical activity with health in children ages 2 to 5 years [1]. At that time, we concluded that the scientific evidence was too weak to determine how much physical activity a preschooler needs. Moreover, there are only a small number of longitudinal studies using accelerometers to measure physical activity of preschoolers (e.g. [14-17]), and none of these have linked physical activity levels with health outcomes such as obesity, health-related fitness, or vascular structure and function.

The goal of the Health Outcomes and Physical activity in Preschoolers (HOPP) study is to describe how the prevalence and patterns of physical activity in preschoolers are associated with a broad spectrum of health outcomes. This longitudinal, observational study will follow 3-, 4-, and 5-year-old boys and girls for 3 years with annual assessments of physical activity, body composition, health-related fitness, vascular structure and function, and health-related quality of life, along with motor skills and parents’ beliefs about physical activity for their preschooler.

Specifically, our objectives are:

1) To use high-frequency accelerometry to assess physical activity prevalence and patterns in 3- to 5-year-old children. We hypothesize that how preschoolers accumulate their physical activity (pattern) will be as important as how much physical activity (prevalence) they accumulate, in terms of positive relationships with health outcomes;

2) To determine the relationships between health outcomes and physical activity in preschoolers throughout the preschool years. We hypothesize that those children who engage in more bouts of moderate-to-vigorous physical activity (MVPA) and who are able to maintain participation in MVPA over the 3-year study period will have more favourable outcomes for body composition, health-related fitness, vascular structure and function, and health-related quality of life;

3) To examine factors relevant to support a physically-active lifestyle of preschoolers. We hypothesize that motor skills and parents’ beliefs will be strong predictors of physical activity at each annual assessment, but strongest at younger ages.

## Methods/design

### Study design

The HOPP study is a prospective cohort study to evaluate the relationship between physical activity and health outcomes. At each assessment, we also examine potentially modifiable factors (parents’ beliefs and motor skills) that could serve as targets for future interventions to influence preschooler physical activity. Data collection occurs at study visits through questionnaire and non-invasive physical measurements. All study procedures have been pilot tested and modified as necessary for the main study [18-21]. The Hamilton Health Sciences/Faculty of Health Sciences Research Ethics Board has provided ethical approval to the study.

### Participants

Participants in the HOPP study are recruited from the south-central Ontario region, including the City of Hamilton and surrounding area. Eligibility for participation is based on medical criteria and not demographic factors. Children with a physical disability or known motor delay or a diagnosed medical condition (e.g., diabetes, arthritis, asthma, cystic fibrosis) are ineligible. Our decision to exclude children with a medical condition is based on the need to first understand the associations between physical activity and health outcomes in typically developing preschoolers – an understanding that is far from complete.

#### *Recruitment Strategy*

We have implemented a community-based recruitment strategy. This strategy works with the Ontario Early Years Centres (Government-funded centres offering services related to early childhood development), preschools and daycare centres in the City of Hamilton and surrounding area, and local school boards. To improve participant retention, we provide parents with yearly reports of key results and study progress. When we send the report, we request information about re-location, etc., and ask about any foreseeable changes to their address.

### Study protocol

Participants and their families follow the same procedures at each annual assessment. We conducted a number of smaller pilot studies [18-21] to develop the protocol, which consists of 2 laboratory visits and 7 consecutive days of physical activity monitoring. Parents visit our laboratory where the study is explained in detail and any questions are answered; written informed consent is then obtained from the parent. Parents then provide demographic information and complete questionnaires. The remainder of this first visit is used to assess body composition, aerobic fitness, short-term muscle power and motor skills. At the end of visit 1, an accelerometer to monitor physical activity is given to the child and their parents are provided detailed instructions on its use for the next 7 days. For their final visit, indices of vascular structure and function are assessed and the accelerometer returned.

### Measurements

#### *Physical activity*

Accelerometers are now quite common in physical activity research. The Actigraph is the most commonly used accelerometer to assess physical activity in children. It has excellent intra- and inter-instrument reliability across a wide range of accelerations [22], and has been validated for use in 3- to 5-year-old children [23]. We use the Actigraph GT3XE activity monitor which weighs 27 g with dimensions of 3.8 × 3.7 × 1.8 cm (about the size of a matchbox) and measures accelerations ranging in magnitude from ~0.05 to 2 G’s. The acceleration signal is sampled and digitized by a 12-bit Analog to Digital Converter at a rate of 30 Hz. The signal then passes through a digital filter that band-limits the accelerometer to the frequency range of 0.25 to 2.5 Hz, which detects normal human motion and rejects motion from other sources. The GT3XE is secured to the child by a belt worn around the waist. The Actigraph GT3XE accelerometer is ideal for the HOPP study because it can program short epochs (sampling intervals) allowing more accurate measurement of children’s physical activity behaviour [18]. We use a 3-s epoch because this duration approximates the mean and median duration of vigorous activity bouts observed in young children [24] and, based on our pilot work, accurately captures physical activity intensity [25]. Over the 7 days of monitoring, the device is only removed when the child is sleeping or when exposed to water (e.g., bathing or swimming) since it is not waterproof. Parents keep a log book to record the time of day when the accelerometer is placed on the child and taken off and the reasons why, including any activities that occur during this time (e.g., a nap or swimming lessons). In our pilot work, we achieved a mean monitoring (wearing) time of 11 h per day. The data are visually inspected and only discarded when corresponding to a time the child was not wearing the accelerometer, based on log books.

*How do we determine physical activity prevalence and pattern?* Accelerometer output (counts) is usually analyzed according to cut-off values or thresholds reflecting different intensities of physical activity, and there are numerous thresholds to choose from. We use cut-off values developed and validated specifically with healthy 3- to 5-year-old children using Actigraph accelerometers [23]. These are: 420 counts/15 s (moderate intensity) and 842 counts/15 s (vigorous intensity). Because we use 3-s epochs, we divide these counts by 5 to establish counts per 3 s, as previously published [18]; thus, the physical activity intensity categories used in the HOPP study are: sedentary (<8 counts/3 s); light (≥8 but <84 counts/3 s); moderate (≥84 but <168 counts/3-s); and vigorous (≥168 counts/3-s). To determine physical activity prevalence, we calculate each intensity category as minutes per day and as % of time when the accelerometer was worn, to account for individual differences in monitoring time. To determine physical activity patterns, we calculate the number of bouts of continuous activity at each intensity category, as previously described [18]. To accommodate these analyses, we developed custom Microsoft EXCELs™ macros.

#### *Health outcomes*

##### *Body Composition*

We perform standard measures of anthropometry, including standing height measured without shoes to the nearest 0.1 cm using a calibrated stadiometer and body mass measured without shoes and in light clothing to the nearest 0.1 kg using a digital scale. A bioelectric impedance analyzer is used to assess body composition while children are in a supine position. Fat free mass (FFM) is calculated using an age-specific equation [26], which has been validated in 4- to 6-year–old children using doubly-labelled water. Percent body fat is calculated as [(Body weight – FFM)/body weight] x 100. As an additional estimate of body fat distribution, we measure waist circumference as a surrogate of central adiposity in preschoolers [27]. Measurements are made in duplicate at 3 different locations – uppermost lateral border of the iliac crest, midpoint between the lower margin of the last palpable rip and the top of the iliac crest, and 4 cm above the umbilicus. In a subset of participants (n = 100), we are also assessing body composition using dual energy x-ray absorptiometry (DXA). Participants are scanned in a supine position using a Hologic QDR 4500A scanner. In addition to total body analysis, we perform regional lean mass and fat mass calculations with the pediatric software (Version 12.3) accompanying the DXA scanner.

##### *Health-related fitness*

Aerobic fitness is assessed on a treadmill using the Bruce protocol, a progressive treadmill test, as previously described [28]. Participants are given several minutes of familiarization on the treadmill at a comfortable walking pace. During the test, participants are instructed to hold onto the handrails to assist with coordination, with a researcher positioned directly behind the child to ensure safety. Heart rate (HR) is recorded continuously during the test using a HR monitor (Polar Electro, Kempele, Finland). The test is terminated when the child can no longer keep up with the increasing grade or slope of the treadmill, or by refusal to continue exercising despite verbal encouragement. Following test termination, the child is immediately seated and asked to remain as still as possible. HR is recorded every 30 s during this recovery period and HR recovery is calculated as the difference between HR at the end of exercise and at each time point of recovery, with higher values indicating faster recovery. In our pilot work we found evaluation of HR recovery according to these procedures to be feasible and reliable [19]. To determine short-term muscle power, we developed a 10-s modified Wingate anaerobic cycling test that is feasible and reliable for preschoolers [19]. To establish peak pedalling speed, the child first performs an initial “sprint” by pedaling as fast as they can for ~20 s against only the internal resistance of the ergometer (Pediatric Corival, LODE). Braking force on the ergometer is calculated as 0.55 N·m·kg^-1^ body mass. The child is instructed to pedal as fast as they can with a “ready… set… go” signal from the investigator. When 80 % of the peak pedalling speed is reached, the braking force is applied. During the test, the child is encouraged to keep pedalling as fast as he/she can. Peak power output is taken as the highest power output during the test; mean power output is taken as the average power output over the 10 s.

##### *Vascular structure and function*

Arterial stiffness and wall thickness are assessed using non-invasive methods, as previously described in our pilot work [21]. Participants rest in the supine position and single-lead electrocardiography (ECG) is continuously recorded. After ~10 min of rest, longitudinal images of the carotid artery are acquired using b-mode ultrasound. A linear array transducer is positioned at the lateral side of the neck (Vivid-q, GE Medical Systems, Horten, Norway) and ECG-gated images representing 10 consecutive heart cycles (sampling frequency: 23 frames/s) are obtained. To determine carotid artery stiffness, two additional 10-beat clips are recorded simultaneous to acquisition of carotid artery blood pressure waveforms on the opposite common carotid artery, using a hand-held tonometer (model SPT-301, Millar Instruments Inc., Houston, TX). The carotid pressure waveforms are calibrated to discrete measures of brachial blood pressure using an automated device (Dinamap Pro 100, Critikon LCC, Tampa, FL.). Images are digitally stored for off-line analyses using an automated edge tracking system [AMS II (Artery Measurement System) Image and Data Analysis, Tomas Gustavsson], which provides measurements of arterial borders (intima to media and internal diameter) on all acquired frames. Intima-media thickness is assessed at the far-wall on end diastolic frames and carotid artery stiffness (distensibility) is calculated as: [(maximum diameter – minimum diameter)/(carotid pulse pressure x minimum diameter)].

Arterial stiffness is also determined using “whole body” pulse wave velocity in a large segment of the arterial tree (heart to dorsalis pedis artery in foot). A photoplethysmographic probe (MLT1020PPG, ADInstruments, Colorado Springs, CO, USA) is placed on the skin over the dorsalis pedis artery. The time delay from ventricular depolarization (R-spike on ECG) to the arrival of the pulse wave at the artery is calculated and divided into the measured body surface distance between these sites (i.e., suprasternal notch to foot). Following supine measurements, children are seated and automated cuff measurements of brachial blood pressure are obtained after 2–3 min of seated rest. We have previously established the reliability of these measures in 3- to 5-year-old children [21].

##### *Health-related quality of life*

We measure health-related quality of life to capture domains of health such as emotional and social functioning, which are relevant to early child development. To determine the association between physical activity and health-relate quality of life, parents/guardians complete the 23-item PedsQL™ 4.0 Generic Scale. This instrument measures: Physical Functioning (8 items); Emotional Functioning (5 items); Social Functioning (5 items); and Daycare/School Functioning (5 items). Excellent feasibility, reliability, and validity for this instrument have been demonstrated on a sample of 1,257 three-year-olds, 1,198 four-year-olds and 984 five-year-olds [29].

#### *Additional measures*

In addition to the above measurements, parents provide demographic (ethnicity, family income, childcare arrangements, etc.) and medical (child’s birth weight, duration of breastfeeding, etc.) information as well as information on their child’s participation in organized and unorganized activities, sports, and screen time. Other measures include:

##### *Dietary intake*

To help interpret the relationship between physical activity and health outcomes, particularly body composition, dietary intake is also assessed. Parents record their child’s diet for 2 days during the week (Monday-Friday) and 1 day on the weekend (Saturday or Sunday). Food and drinks consumed are recorded for all meals and snacks, with quantities, brand names, and preparation methods included where appropriate. Information is entered into the ESHA Food Processor software (Version 10.8, Salem, OR) and analyzed.

##### *Children’s Behaviour Questionnaire*

To assess the child’s temperament, parents complete the Children’s Behaviour Questionnaire (very short form), which determines temperament across three broad dimensions: surgency, negative affect, and effortful control [30,31].

##### *Motor Skills*

Motor skills are being developed during the early years [32], and typically developing preschoolers with better motor skill proficiency tend to display higher levels of physical activity [33-37], particularly participation in vigorous physical activity [37]. We measure motor skills using 2 tests. The first test is the Peabody Developmental Motor Scales-2. This is a criterion and norm-referenced scale composed of six subtests that measure interrelated motor abilities that develop early in life. It is designed to assess gross and fine motor skills of children from birth through 5 years of age; we use only subtests for gross motor skill assessment. The second test is the short form of the Bruinitisky-Oserestky Test of Motor Proficiency (BOT-2). The BOT-2 short form is composed of 14 items and provides a total motor composite score across 4 areas: fine manual control, manual coordination, body coordination, and strength and agility. The BOT-2 is designed to identify individuals with mild to moderate coordination deficits [38]. It is validated for use in 4–21 year olds; thus, it is not used at the initial assessment of 3-year-olds. In addition, all children complete two BOT-2 tasks that are not included in the short form: 50 ft shuttle run and standing long-jump.

##### *Parents’ Beliefs about Physical Activity for their Preschooler*

Using an approach based on the theory of planned behaviour [39] encompassing parental attitudes regarding their child's physical activity, social normative beliefs, perceived control/self-efficacy, and intentions, we developed a questionnaire to assess parents/caregivers’ beliefs about physical activity for their preschooler. The theory of planned behaviour has been used extensively in prospective and intervention studies of health related behaviours including physical activity [40], with variables in the theory accounting for 29 % of the variance in physical activity behaviour. Following recommendations by Ajzen [41], we gathered pilot data from a sample of parents (n = 26), assessing their behavioural, normative, and control beliefs regarding their preschool children’s participation in moderate-vigorous intensity physical activities. Content analysis of those data was carried out and results used to develop measures of parents’ attitudes (6 items), subjective norms (7 items), perceived behavioural control (5 items) and behavioural intentions (3 items). We also examine parents' self-efficacy regarding their abilities to provide opportunities for their children to be active. Using guidelines by Bandura [42], a behaviourally-specific self-efficacy measure (9 items) was constructed based on pilot data identifying common barriers and scheduling issues relevant to preschoolers’ physical activity. Our decision to focus on parents’ beliefs is consistent with their own reporting that their beliefs about physical activity probably influence that of their preschooler [43]. Parent physical activity levels are also assessed using the International Physical Activity Questionnaire for 15–69 year olds (Short Form).

### Data analyses

We are not interested in simply describing changes in physical activity over a 3-year period in young children. Rather, the novelty in the HOPP study is in describing how the prevalence and patterns of physical activity in preschoolers are associated with health outcomes, and whether the strength of these associations changes as preschoolers advance through the early years. In addition to performing standard linear regression analyses for each health outcome with physical activity (i.e., cross-sectional analyses), we propose to assess the associations between each key health outcome and physical activity using mixed-effects modeling to conduct longitudinal analyses, thereby taking full advantage of the richness of our data set. In addition to characterizing and testing the average pattern of change in preschoolers of different physical activity levels, the mixed-effects analyses provide for both random- and fixed-effects of the parameters of change that correctly account for the repeated measurements within the same child. In effect, individual change curves are predicted for each child and the degree of individual variations are estimated. We can then include other available information about the children to predict or explain variations in both initial status and subsequent change. These predictors may include both important baseline covariates such as gender and ethnicity, but also potentially-modifiable factors (e.g., parents’ beliefs, motor skill proficiency) that are hypothesized to influence the risk of unhealthy trajectories.). Similar analyses can be performed for each health outcome plotted as the trajectory of interest.

### Sample size estimation

Based on our pilot studies, the association between daily minutes of physical activity and various health outcomes demonstrated effects size (R^2^) of at least 0.11, indicating medium effect sizes according to Cohen, as referenced by Green [44]. Thus, for regression analyses to be completed at each year of assessment, based on medium effect sizes, Green [44] recommends a sample size of 50 + 8(m) (where m = number of predictors). With up to 6 predictors in a comprehensive model (e.g., physical activity variable, sex, parents’ beliefs, motor skills, socio-economic status, and child temperament), a sample size of 100 in each age group would provide at least 80 % power for those statistical tests. Thus, with 3 age groups (3, 4, and 5 years) at baseline, 300 children are needed. Assuming an attrition rate of 10 % per year, our goal is to recruit 400 children. We also wish to ensure the adequacy of this sample with respect to the linear mixed-effects analysis. Sample size calculations for this purpose are complex, and generally require too many assumptions to be practical. It is more typical to calibrate the size of the sample to the complexity of the analysis by ensuring that the ratio of observations to the expected number of estimated parameters in the analysis is at least 5–10. For this longitudinal analysis, we will have 400 children assessed on 3 occasions, yielding 1,200 observations for all variables in the analysis, and a ratio well above the 5–10 recommendation, allowing for the potential of missing data.

## Discussion

The HOPP study is different from other longitudinal studies of physical activity in preschoolers because it will determine the relationship between being active and being healthy. Many preschoolers worldwide are suffering the obesity epidemic, which may be accelerating the onset of cardiovascular disease. In recent years, several correlates or potential determinants of preschooler physical activity have been identified [1,12,13], yet we know very little about the health benefits of physical activity during the early years [1]. The goal of the HOPP study is to provide evidence linking physical activity and health outcomes in preschoolers, and to examine these relationships over time.

Although the goal of the HOPP study is to determine the relationship between physical activity and health outcomes during the early years, it is important to assess factors that could serve as targets for future interventions to influence preschooler physical activity. For example, the dependence of young children on their parents demands an assessment of the extent to which parents’ beliefs about physical activity influence their preschooler’s physical activity levels. Preschoolers are likely to develop physical activity-related cognitions such as attitudes; however, through their belief-based actions, parents are in a position of strong control to enable or impair preschooler physical activity behaviours [45]. Motor skill proficiency is also correlated with physical activity levels among preschoolers. However, the causal relation between motor skill proficiency and physical activity remains unclear; are preschoolers more active because of better motor skills or do they have better motor skills because they are more active? The longitudinal design of the HOPP study will be able to address some of these issues.

We also propose that understanding the pattern of physical activity may be as important as how much physical activity preschoolers accumulate on a daily basis. Physical activity patterns can be described as how physical activity is accumulated (i.e., frequency and duration of activity). To accurately measure physical activity patterns of preschoolers, it is essential to utilize objective methods with the high-frequency recording capacity needed to capture their movement behaviour [18]. A novel aspect of the HOPP study is the use of high-frequency accelerometry, which will allow us to explore relationships between the frequency and duration of bouts of physical activity at different intensities with a broad range of health outcomes. For example, total physical activity, representing overall energy expenditure, may be more related to adiposity whereas bouts of moderate-to-vigorous physical activity, representing greater cardiovascular stress, may be more related to vascular structure and function.

The HOPP study will be able to characterize how the prevalence and patterns of physical activity of preschoolers are associated with key health outcomes throughout the early years. We plan to disseminate knowledge to the public and among stakeholders responsible for early child development. We expect our findings will hold the potential to shape public health policy for active living during the early years, a critical time to develop these behaviours and for preschoolers to focus on the ‘business’ of growing up.

## Abbreviations

HOPP = Health outcomes and physical activity in preschoolers; MVPA = moderate-to-vigorous physical activity; FFM = fat-free mass; DXA = dual energy x-ray absorptiometry; HR = heart rate; ECG = electrocardiography; BOT-2 = Bruinitisky-Oserestky test of motor proficiency – Version 2.

## Competing interests

There are no competing interests.

## Authors’ contributions

BWT conceived and designed the study and drafted the manuscript. NAP was involved in drafting the manuscript. MJM contributed to the design of the study and was involved in drafting the manuscript. SRB contributed to the design of the study and was involved in drafting the manuscript. JC contributed to the design of the study and was involved in drafting the manuscript. All authors read and approved the final manuscript.

## Funding

The HOPP study is funded by the Canadian Institutes of Health Research (CIHR Award #: MOP 102560). BWT is supported by a CIHR New Investigators Award. JC is supported by an endowed professorship through the department of Family Medicine, McMaster University.

## Pre-publication history

The pre-publication history for this paper can be accessed here:

http://www.biomedcentral.com/1471-2458/12/284/prepub
